# The World Needs 6 Million More Nurses: What Are We Waiting For?

**DOI:** 10.4269/ajtmh.20-0451

**Published:** 2020-05-15

**Authors:** Stella Aguinaga Bialous, Kimberly Baltzell

**Affiliations:** School of Nursing, University of California San Francisco, San Francisco, California

The WHO declared that 2020 would be the Year of the Nurse and the Midwife in recognition of the “vital role” nurses and midwives play in the health system.^1^ The celebration was designed to coincide with the 200th birthday of Florence Nightingale. Considered the founder of modern nursing, Nightingale also played a pivotal role in managing infectious diseases.

Modern nursing began with the response of untrained individuals, usually women, to the urgent need of those injured in the Crimean and U.S. Civil wars and, later, those sickened by the 1918 Spanish flu. As we face this century’s virus pandemic, nurses have once again answered the call. Public and media attention have focused on nurses and other healthcare professionals caring for those affected at great personal risk to themselves.

Healthcare focus on the pandemic is needed, but healthcare shortages and deficiencies and nurses’ redirection from non–COVID-19 patients put populations and patients at risk. Nurses and midwives are needed to provide safe, evidence-based care to all people. People are still having babies, heart attacks, and dementia. Nurses are still needed to provide childhood vaccines and cancer care, lifesaving tuberculosis and HIV medications, and hospice care for those with terminal illness. This is essential, not elective, care. The full impact of essential care delays is yet to be fully known.^2^

Every day nurses answer a call to service. Nurses see seven to eight of every 10 patients seeking health care in the world. The first ever WHO State of the World’s Nursing report^3^ confirms the critical role of nursing in achieving universal health coverage. The report also documents the shortage of nurses; 80% of the nursing workforce works in countries with 50% of the world’s population. To put it in another way, the African continent has one percent of the world’s healthcare workers (most of whom are nurses) but 25% of the global burden of disease. This imbalance is evident in places such as sub-Saharan Africa, where nurses are often responsible for important care such as initiation of antiretroviral therapy for HIV-infected patients. The significance of this role is confirmed by data. For example, when HIV-infected children are diagnosed and treated early by providers, primarily nurses, mortality is reduced by more than 75%.^4^

Nurses and midwives with advanced practice degrees and specialized education deliver safe and cost-effective care in many countries. However, half of the world’s countries do not have educational pathways for advanced practice.^3^ In particular, COVID-19 has illuminated how a lack of specialized critical care nurses influences patient outcomes. Critical care nurses are often responsible for ventilator support, maintaining adequate intravenous access, assisting in intubation and extubation, titrating complex medications, and playing the role of family and palliative care support for seriously ill patients, among many other tasks. Their competence in these roles is widely recognized. The University of Southern California Medical Center surgical residents recently underwent training in critical care nursing skills to improve their ability to care for COVID-19 patients. This training was initiated when surgical residents filling in for critical care nurses demonstrated a dearth of skills needed in the intensive care unit.^5^

Nurses have played critical roles in the successes of the President’s Emergency Plan For AIDS Relief, the CDC, the U.S. military, and more.^6^ Immunizations, considered one of the 10 greatest public health achievements during the twentieth century, are largely delivered by pediatric nurses,^7^ and nurses are pivotal to the success of immunization campaigns.^8^ And whereas access to appropriately trained midwives has been shown to improve both maternal and neonatal outcomes, lack of investment in quality midwifery education, poor regulation, and poor working conditions continue in many places.^3,9^ In general, nurses are like oxygen: largely taken for granted, it is only in their absence that their vital importance is felt.

Nurses are pivotal to reaching WHO sustainable development goals.^3^ And yet, for decades, we have known that there is a global shortage of nurses. Worldwide, there is a shortage of almost 6 million nurses, according to the WHO.^3^ In the United States alone, we will need more than 1 million more nurses by 2026, according to the U.S. Department of Labor.^10^

The current COVID-19 pandemic has been exacerbated–and we will continue to see more of this if the virus takes off in Africa and Latin America–by insufficient health system capacity. So when the pandemic is eventually controlled, let us celebrate nurses and midwives. But then let us give them the gift of a serious policy discussion on how we will educate and supply more nurses to address the ongoing global needs. And let us make sure they have the tools they need to do the job they love.

Nurses all over the planet are doing their job, rejoicing in patients’ recoveries, and supporting families in their grieving. They deserve no less than to have the future of their profession be taken seriously in the Year of the Nurse and Midwife. Two hundred years after Florence Nightingale, it is about time.
